# Editorial: Advances in Mechatronics and Biomechanics Towards Efficient Robot Actuation

**DOI:** 10.3389/frobt.2019.00019

**Published:** 2019-03-26

**Authors:** Jörn Malzahn, Navvab Kashiri, Monica A. Daley, Nikos Tsagarakis

**Affiliations:** ^1^Humanoids and Human Centred Mechatronics Lab, Department of Advanced Robotics, Istituto Italiano di Tecnologia, Genova, Italy; ^2^Structure and Motion Laboratory, Royal Veterinary College, London, United Kingdom

**Keywords:** energy efficient actuation, actuator design, actuator control, energetics of human locomotion, energetics of animal locomotion, bioinspired control, bioinspired design

Biological systems such as animals and humans can be energetically autonomous for several days and some species endure even longer. Today's robotic systems aim to match the physical capabilities of their biological counterparts in terms of strength, agility, and dexterity, yet fail to display untethered operating times any longer than few hours. Changing this situation is challenging and likely requires fundamental shifts in the current paradigms for robot designs.

Today, new and promising paradigms have evolved from the recent advances at the interface of robotics and biomechanics. Robotics has become a prominent tool for validating and testing biomechanical models of animal locomotion, allowing direct testing of hypotheses about the mechanisms behind locomotion in nature. The physical implementation of the biomechanical models in robotic systems allows quantitative evaluation of theory and comparison between replicated movement and force patterns to those observed in biology. This approach has recently advanced our understanding of nature's locomotion principles, in particular for the integration of neural control and actuation for agile locomotion.

These new biomechanics insights are now being turned into novel mechatronic paradigms and solutions for dynamic and efficient walking robots and robust and lightweight robotic prostheses and orthoses. Conventional robot designs have been dominated by chains of rigid links with high transmission ratio non-backdrivable joints. The integration of intrinsically compliant components into robot designs has been researched for more than two decades now. However, we have only recently gained sufficient understanding to effectively exploit passive springs and intrinsically backdrivable low transmission ratio actuators for low inertia designs, with force sensing and control for efficient and impact resilient actuation. Impact resilience is especially important because impacts on the mechanism represent normal operating conditions for legged locomotion in unstructured environments and realistic terrain. Further insights on neural control in biological systems continues to spawn new paradigms for hierarchical and hybrid local/distributed control architectures. These bio-inpsired control advances help to tackle the task complexity involved in enabling robots to robustly perform in real-world scenarios outside the lab. Recent findings in this realm suggest that, in some cases, control loops need not run at high rates (> 1 kHz) to achieve robust performance. Sometimes closing a loop, with the associated delays and efforts to ensure stability, might not be advisable at all. Instead feedforward actions along with carefully shaped natural dynamics can lead to simpler, more robust and efficient solutions.

The present Research Topic joins research efforts from the robotics, control, and biomechanics communities toward novel robot design and control paradigms. The joint efforts included the organization of a physical work meeting in the form of a joint workshop on the energetic economy of robotics and biological systems during the International Conference on Intelligent Robots and Systems (IROS) on September 24, 2017^1^.

Given the interdisciplinary nature of the Research Topic, it is organized under the sections “Humanoid Robotics” and “Robotic Control Systems” within *Frontiers in Robotics and AI* as well as the “Bionics and Biomimetics” section with *Frontiers in Bioengineering and Biotechnology*. The collection of articles in this Research Topic captures a wide-angled perspective on the current state-of-the-art in robotic as well as biodynamic actuation modeling, analysis, design and control toward enhanced robot energy efficiency.

The wide-angled perspective is initialized with an overview article by Kashiri et al. on fundamental principles of locomotion inspired from natural biological systems, and principles for the design and control for efficient robot locomotion. As a foundation, Lee and Harris present and discuss a framework for quantitative evaluation of locomotion efficiency in animals and robots. The Research Topic further elaborates on some of the locomotor principles outlined in the overview with dedicated articles on minimally actuated walkers (Schroeder and Bertram) and articulated compliant parallel actuation arrangements (Roozing).

Nature demonstrates elegant solutions to minimize the inertia of moving limbs through remote tendon driven actuation. Several articles in the Research Topic are devoted to the potential and the challenges encountered during the technical implementation and application of such principles (Grosu et al.; Tian et al.; Wagner and Emmanouil).

Urbina-Meléndez et al. model and study how observability and state-estimation in balancing skills depends on the physical location of sensory organs. Nguyen et al. investigate the activation and response behavior of biological muscles. Engineers seeking to find suitable target functions for actuator designs or optimal sensor placements in their robots can greatly benefit from these insights, which emerge from millions of years of natural evolutionary design iterations.

Nature seems to effortlessly manage complexity, not only concerning growing and self-regenerating biomechanics, but also considering efficient and effective control of millions of cells, sensors and muscle fibres. In this Research Topic Groothuis et al. address this topic in the context of energy aware control across distributed control hierarchies as observed in biology and—with the aim to manage system complexity—also applied to technical systems (Barasuol et al.).

Apart from mobile and legged robots, the direct beneficiaries of the mutual insights in biodynamics and robotics engineering are humans. This Research Topic includes examples of robotic rehabilitation systems (Ghannadi et al.) as well as assistive robotic devices such as passive and active prostheses (Jeffers and Grabowski; Tahir et al.) to improve or recover the quality of life.

The editors hope that the results presented in this Research Topic will catalyze the advent of new robots and assistive robotic devices with enduring energetic autonomy, which will help us tackle the upcoming societal and economic challenges. At the same time the presented works report unique and invaluable experimental data that help verifying and refining theories as well as hypotheses striving to understand the nature of the species living on our planet (Nguyen et al.; Urbina-Mele'ndez et al).

## Author Contributions

All authors listed have made a substantial, direct and intellectual contribution to the work, and approved it for publication.

### Conflict of Interest Statement

The authors declare that the research was conducted in the absence of any commercial or financial relationships that could be construed as a potential conflict of interest.

## Footnotes

^1^https://energeticeconomyrobotics.wordpress.com/

